# Opening Pandora’s box: A meta-ethnography about alcohol use in pregnancy from midwives’ and other healthcare providers’ perspectives

**DOI:** 10.18332/ejm/166189

**Published:** 2023-07-03

**Authors:** Bente Dahl, Aud Johannessen, Terese Bondas

**Affiliations:** 1 Faculty of Health Sciences, University of Stavanger, Stavanger, Norway; 2 Centre for Women’s, Family and Child Health, Faculty of Health and Social Sciences, University of South-Eastern Norway, Borre, Norway; 3 Norwegian National Centre for Ageing and Health, Vestfold Hospital Trust, Tønsberg, Norway

**Keywords:** alcohol, pregnancy, midwife, antenatal care, healthcare provider, meta-ethnography

## Abstract

**INTRODUCTION:**

Alcohol consumption has increased in recent years, including among women of childbearing age. A woman’s alcohol intake during pregnancy is linked to complications and injuries in the newborn, and the risk of the child being harmed by the mother’s alcohol use increases in proportion to the amount of alcohol she consumes. This meta-ethnography aims to explore midwives’ and other healthcare providers’ experiences of screening pregnant women for alcohol use in pregnancy and counselling them on the subject.

**METHODS:**

A systematic literature search in CINAHL, Maternity & Infant Care, MEDLINE, and Scopus was conducted in August 2021 and updated in January 2023. The CASP checklist was used to assess the included articles and meta-ethnography was used to synthesize the data.

**RESULTS:**

Fourteen qualitative studies were included. In the synthesis, we use the metaphor of Pandora’s box to deepen our understanding of the topic. We found that some healthcare providers tiptoe around the box, not wanting to face the consequences and responsibilities of asking women about their alcohol use. Others refuse or are reluctant to open the box because they lack knowledge about screening and counselling. Some eventually open the box, understanding the importance of establishing a trusting relationship to address alcohol use and seeing the need for knowledge and screening tools.

**CONCLUSIONS:**

Healthcare education has the important task of ensuring that healthcare personnel have sufficient evidence-based knowledge about alcohol use in pregnancy. In the future, a health-promoting, tailored approach offering women in pre-pregnancy and early pregnancy sufficient evidence-based information should be implemented.

## INTRODUCTION

The mean age of first-time mothers has increased worldwide, and the average age for giving birth is ≥30 years in most OECD countries^1^. This may imply that alcohol consumption before pregnancy is becoming increasingly common^2^. There has been an increase in alcohol consumption in recent years, including among women of childbearing age^3^, and 9.8% of women globally continue to consume alcohol in pregnancy (AIP)^4^. A study mapping alcohol use in pregnancy in many European countries found an overall prevalence of alcohol consumption of 15.8%. The study reported that women in the United Kingdom, Russia and Switzerland had the highest consumption, whereas women in Norway, Sweden and Poland had the lowest^5^.

The World Health Organization (WHO) has reported that elevated alcohol consumption is linked to more than 200 health conditions^6^. Most women change their alcohol use when they become pregnant, the baby’s health being the key reason for abstaining or reducing consumption. However, research underscores that high maternal age and high alcohol intake before pregnancy constitute risk factors for use of AIP^7^. Alcohol intake can be linked to complications and injuries in the newborn, and the risk of the child being harmed by the mother’s alcohol use increases in proportion to the amount of alcohol she consumes^8^. Fetal alcohol spectrum disorder (FASD) is an umbrella term for conditions that seriously impact a fetus whose mother has been using AIP^9^. It is a worldwide public health concern and consists of several diagnostic entities, including fetal alcohol syndrome (FAS)^10^. An estimated one in thirteen women having consumed AIP gives birth to a child suffering from FASD, resulting in 630000 children born with FASD globally every year^11^.

Healthcare providers (HCPs), including midwives, have an important task in mapping women’s alcohol use before pregnancy and giving advice on alcohol use during pregnancy. Drinking in pregnancy is an emotional issue and delivering brief interventions concerning alcohol use at the booking of an appointment may be challenging^12^. However, many women experience pregnancy as a time when they are motivated to give their unborn children the best start in life. Most women attend antenatal care (ANC), and therefore this is an excellent period for health promotion and possible lifestyle change^13,14^. Health promotion enables people to increase control over their own health^15^. A wide range of social and environmental interventions are designed to benefit and protect people’s health and quality of life by addressing and preventing the root causes of ill health, not just focusing on treatment and cure. Screening and surveillance activities can be regarded as health-promoting aspects of antenatal care, like screening for alcohol use, providing evidence-based information about consequences of alcohol use on the fetus, and using brief interventions to encourage behavior changes. A review^16^ concluded that screening for alcohol use in pregnancy should be mandatory and implemented consistently. Moreover, HCPs should have the capacity to inform and advise pregnant women about the risks of AIP. Chang^17^ refers to the WHO^18^ guidelines for the identification and management of substance use during pregnancy, listing various potential screening measures. Baron et al.^19^ found that having trusting relationships with women allowed midwives to talk about AIP. Thus, midwives should err on the side of providing too much rather than too little information about alcohol use. Nevertheless, van der Wulp et al.^20^ found that midwives doubted the importance of alcohol advice in pregnancy. Integration and synthesis of knowledge about health promotion in antenatal care related to alcohol use provided by HCPs is essential. Thus, this meta-ethnography study aims to explore midwives’ and other HCPs’ experiences of screening pregnant women for and counselling them about alcohol use in pregnancy.

## METHODS

### Design

Meta-ethnography is an interpretative methodology for qualitative evidence synthesis^21^. It has become an influential and increasingly popular methodology in health and social care research, drawing on Geertz’s^22^ concept of ‘thick descriptions’ as well as Turner’s^23^ theory of sociological understanding as ‘translation’. Meta-ethnography has the potential to produce new interpretations, models or theories. We conducted the study according to the seven overlapping and iterative phases of meta-ethnography described by Noblit and Hare^24^ (Table 1) and the eMERGe metaethnography reporting guidance^21^ (Table 2). The guidance consists of 19 reporting criteria, covering all phases of the meta-ethnographic method.

**Table 1:** Phases in meta-ethnography^24^

*No.*	*Phases*
1	Getting started, identifying the topic of the study, and defining the aim.
2	Deciding what is relevant to the initial interest including relevant studies, describing search strategy and criteria for inclusion and exclusion.
3	Reading the studies, noting studies’ interpretative metaphors through repeated readings.
4	Determining how the studies are related, determining the relationship between the studies by creating a list of key metaphors (themes, concepts, phrases, ideas) and assessing whether the relationships are reciprocal (i.e. findings across studies are comparable), refutational (findings stand in opposition to each other) or represent a line of argument.
5	Translating the studies into one another, comparing metaphors and their interactions within single studies and across studies, and at the same time protecting uniqueness and holism.
6	Synthesizing translations, creating a new whole from the sum of the parts, enabling a second-level analysis.
7	Expressing the synthesis, finding the appropriate form for the synthesis to be effectively communicated to the audience.

**Table 2:** The eMERGe meta-ethnography reporting guidance^21^

*Phases*	*Criteria headings*	*Reporting criteria*	*Pages*
**Phase 1: Selecting meta-ethnography and getting started**	**Introduction** 1. Rationale and context for the meta-ethnography	Describe the gap in research or knowledge to be filled by the metaethnography and the wider context of the meta-ethnography.	1-2
	2. Aim(s) of the meta-ethnography	Describe the meta-ethnography aim(s).	2
	3. Focus of the meta-ethnography	Describe the meta-ethnography review question(s) (or objectives).	2
	4. Rationale for using metaethnography	Explain why meta-ethnography was considered the most appropriate qualitative synthesis methodology.	2
**Phase 2: Deciding what is relevant**	**Methods** 5. Search strategy	Describe the rationale for the literature search strategy.	2-3
	6. Search processes	Describe how the literature searching was carried out and by whom.	3
	7. Selecting primary studies	Describe the process of study screening and selection, and who was involved.	3-4
	8. Outcome of study selection	Describe the results of study searches and screening.	4
**Phase 3: Reading included studies**	**Methods** 9. Reading and data-extraction approach	Describe the reading and data-extraction method and processes.	4
	**Findings** 10. Presenting characteristics of included studies	Describe characteristics of the included studies.	4
**Phase 4: Determining how studies are related**	**Methods** 11. Process for determining how studies are related	Describe the methods and processes for determining how the included studies are related: a) Which aspects of studies were compared; and b) How the studies were compared.	4-5
	**Findings** 12. Outcome of relating studies	Describe how studies relate to each other.	4-5
**Phase 5: Translating studies into one another**	**Methods** 13. Process of translating studies	Describe the methods of translation: a) Describe steps taken to preserve the context and meaning of the relationships between concepts within and across studies; and b)Describe how the reciprocal and refutational translations were conducted.	4-5
	**Findings** 14. Outcome of translation	Describe how potential alternative interpretations or explanations were considered in the translations and describe the interpretive findings of the translation.	4-5
**Phase 6: Synthesizing translations**	**Methods** 15. Synthesis process	Describe the methods used to develop overarching concepts (‘synthesized translations’) and describe how potential alternative interpretations or explanations were considered in the synthesis.	5-7
	**Findings** 16. Outcome of synthesis process	Describe the new theory, conceptual framework, model, configuration, or interpretation of data developed from the synthesis.	5-7
**Phase 7: Expressing the synthesis**	**Discussion** Summary of findings	Summarize the main interpretive findings of the translation and synthesis, and compare them to existing literature.	7-15
	Strengths, limitations and reflexivity	Reflect on and describe the strengths and limitations of the synthesis: a) Methodological aspects, for example describe how the synthesis findings were influenced by the nature of the included studies and how the meta-ethnography was conducted; and b) Reflexivity, for example, the impact of the research team on the synthesis findings.	15
	Recommendations and conclusions	Describe the implications of the synthesis.	16

### Inclusion criteria and search strategy

We included empirical studies using qualitative methodologies focusing on midwives’ and other HCPs’ experiences of screening pregnant women for alcohol use in pregnancy and counselling them, published between 2010 and 2021. Articles presenting different perspectives were included when it was possible to separate them, and articles using mixed methods were included if they presented discernible findings from the qualitative part. The articles had to be written in English or one of the Nordic languages and published in a peer-reviewed scientific journal. We excluded quantitative studies, theoretical articles, reviews, Master’s theses, and dissertations.

We conducted an initial scoping search in February 2021. This was followed by a discussion with the team and a revision of key terms before new literature searches were conducted in February and August 2021 (Supplementary file Part 1). An updated search was conducted in January 2023 (Supplementary file Part 1). We included four databases: CINAHL, Maternity & Infant Care, MEDLINE, and Scopus. Medical subject headings and text words used for the searches included: ‘alcohol’, ‘alcohol drinking’, ‘alcohol consumption’, ‘pregnant’, ‘gestation’, and ‘maternal’ combined with ‘qualitative’. Boolean operators were used to combine the terms and truncations were used to ensure a sufficiently broad search. We backtracked reference lists in the included articles. The first author was responsible for carrying out the literature searches and experienced librarians assisted in identifying relevant key terms and performing the searches.

### Search outcome

**Figure 1:**
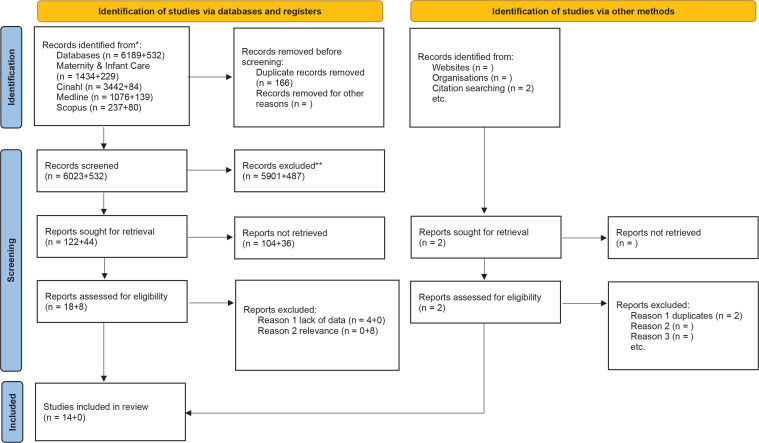
The PRISMA 2020 statement: an updated guideline for reporting systematic reviews^25^

We describe the literature search and screening process in detail in a PRISMA flow diagram^25^ (Figure 1). Article titles were read to remove duplicates and irrelevant publications. The next step involved reviewing abstracts and excluding irrelevant articles or articles employing unsuitable methodologies. The remaining 18 articles were read in full and assessed for eligibility according to the inclusion and exclusion criteria, resulting in the exclusion of four articles due to insufficient data. The reference searches resulted in two articles, but these turned out to be duplicates and were therefore excluded. The updated search did not result in the inclusion of more articles. All authors were involved in the screening process, which began with individual readings and was followed by discussions to reach a consensus on eligibility and inclusion.

### Quality appraisal

To assess the included articles, we used a checklist from the Critical Appraisal Skills Program (CASP)^26^. CASP is designed as a pedagogical tool. For us it served as a systematic reminder of issues related to study quality, including study aim, methodology, recruitment strategy, data collection, reflexivity, ethical issues, data analysis, statement of findings and study value (assessed by 10 questions given in Table 3). We divided the articles that were eligible for inclusion between the authors and assessed them independently. Afterwards, we discussed minor disagreements to reach a final consensus on inclusion. Two articles answered 8 of 10 questions, lacking information about reflexivity and data analysis. Nine answered 9 of 10 questions, lacking information about reflexivity. The remaining three articles answered all 10 questions. We, therefore, considered all articles to have sufficient quality to be included and provide a detailed list of the quality appraisal in Table 3.

**Table 3:** Quality assessment of included studies (CASP)^26^

*Studies*	Assessment questions*									
	*1*	*2*	*3*	*4*	*5*	*6*	*7*	*8*	*9*	*10*
Bagley et al.^29^ (2019)	Y	Y	Y	Y	Y	N	Y	N	Y	Y
Coons et al.^30^ (2017)	Y	Y	Y	Y	Y	N	Y	Y	Y	Y
Crawford-Williams et al.^31^ (2015)	Y	Y	Y	Y	Y	N	Y	Y	Y	Y
Doi et al.^32^ (2014)	Y	Y	Y	Y	Y	N	Y	Y	Y	Y
France et al.^33^ (2010)	Y	Y	Y	Y	Y	Y	Y	Y	Y	Y
Jones et al.^34^ (2011)	Y	Y	Y	Y	Y	N	Y	Y	Y	Y
Loxton et al.^35^ (2013)	Y	Y	Y	Y	Y	N	Y	Y	Y	Y
Oni et al.^36^ (2020)	Y	Y	Y	Y	Y	N	Y	Y	Y	Y
Pedruzzi et al.^37^ (2020)	Y	Y	Y	Y	Y	Y	Y	N	Y	Y
Petersen Williams et al.^38^ (2015)	Y	Y	Y	Y	Y	N	Y	Y	Y	Y
Schölin et al.^39^ (2021)	Y	Y	Y	Y	Y	N	Y	Y	Y	Y
Schölin et al.^40^ (2019)	Y	Y	Y	Y	Y	Y	Y	Y	Y	Y
van der Wulp et al.^41^ (2013)	Y	Y	Y	Y	Y	N	Y	C	Y	Y
Whitehead et al.^42^ (2019)	Y	Y	Y	Y	Y	Y	Y	Y	Y	Y

Y: yes. N: no. C: cannot tell.

* Assessment questions: 1) Was there a clear statement of the aims of the research?; 2) Is a qualitative methodology appropriate?; 3) Was the research design appropriate to address the aims of the research?; 4) Was the recruitment strategy appropriate to the aims of the research?; 5) Were the data collected in a way that addressed the research issue?; 6) Has the relationship between researcher and participants been adequately considered?; 7) Have ethical issues been taken into consideration?; 8) Was the data analysis sufficiently rigorous?; 9) Is there a clear statement of findings?; and10) How valuable is the research?.

### Data extraction, analysis, and synthesis

All authors were involved in the process of repeated reading and data extraction, working in pairs with the first author to discuss possible differences of opinion and overlooked findings. We made use of our experience as qualitative researchers as well as our varied professional (midwife, public health nurse and psychiatric nurse), theoretical and international experience when performing the analysis. We developed a table describing each study’s aim, sample characteristics, research design, data collection, data analysis and key findings to provide context (Table 4) and continued by re-reading the results sections, noting the interpretative metaphors including citations, themes, categories, and concepts in a Microsoft Word table. This enabled us to compare findings across the studies. The next phase, translating the studies into one another, was performed by the first and third authors. This process involved moving back and forth in the data within each study and between the studies without losing sight of the context of the findings. According to Noblit and Hare^24^, it is important to consider the themes, concepts and metaphors in the studies to determine how they are related. They can be directly comparable (reciprocal), they can stand in opposition to each other (refutational), or they can say something about the whole, based on a set of parts (line of argument).

**Table 4:** Characteristics of included studies

*Authors Year Country*	*Aim*	*Sample and setting*	*Research design*	*Methods for data collection and analysis*	*Key findings*
Bagley et al.^29^ 2019 Australia	How perspectives about alcohol and the risks associated with alcohol consumption become non-clinical factors that may influence professional practice responses in relation to fetal alcohol spectrum disorder (FASD).	Pediatricians, psychiatrists, social workers, neuropsychologists, psychologists, speech language therapists, public health nurses, youth workers, mental health nurses, counsellors, family support workers, child protection workers and public health professionals (n=34).	Ethnographic research paradigm	Semi-structured interviews conducted face-to-face or via telephone with participants recruited from professional development workshops on FASD Thematic analysis (Braun & Clarke)	Data revealed four frames of reference practitioners use when thinking about alcohol and risk: reflection on personal experience; experiences of friends, relatives and colleagues; social constructions of alcohol use and misuse; and comparisons to other types of drug use.
Coons et al.^30^ 2017 Canada	To investigate Northern Ontario healthcare students’ attitudes, beliefs, and perceptions as they pertain to alcohol consumption during pregnancy, as well as the recommendations provided to pregnant women.	Participants included students in their final 2 years of school and the total sample (n=21) comprised seven undergraduate medicine students, eight midwifery students, six nurse practitioner students.	Social Constructionism the guiding theoretical study framework	Vignettes and semi-structured interviews Thematic analysis (Braun & Clarke)	Two primary themes related to students’ attitudes concerning alcohol consumption during pregnancy were identified: a) divergent recommendations for different women, based on perceptions of their level of education, culture/ethnicity, and ability to stop drinking; and b) understanding the social determinants of health, including the normalization of women’s alcohol consumption and potential partner violence.
Crawford-Williams et al.^31^ 2015 Australia	To explore the advice that health professionals provide to pregnant women about alcohol consumption; the knowledge of health professionals regarding the effects of alcohol consumption; and their consistency with following the Australian guidelines.	5 health professionals from private practice (one general practitioner), one midwife, and three obstetricians) and five from the public sector (three hospital midwives, and two GPs). Seven participants were female and three were male	Qualitative design	Semi-structured face-to-face interviews Thematic analysis framework (Braun & Clarke)	Health professionals displayed adequate knowledge that alcohol can cause physical and mental difficulties that are lifelong: however, knowledge of the term FASD and the broad spectrum of difficulties associated with alcohol consumption during pregnancy was limited. Although health professionals were willing to discuss alcohol with pregnant women, many did not make this a routine part of practice, and several concerning judgements were noted.
Doi et al.^32^ 2014 Scotland	To explore midwives’ attitudes and practices regarding alcohol screening and ABIs in order to understand why they are relatively underutilized in antenatal care settings compared to other clinical settings.	15 midwives and a focus group with a further six midwifery team leaders (n=21)	Qualitative design	Semi-structured interviews Thematic analysis using Fereday & Muir-Cochrane, Boyatiz and Crabtree & Miller	Midwives were positive about their involvement in the screening and ABI program. However, they were not completely convinced about the purpose and value of the screening and ABIs in antenatal care. In the midst of competing priorities, the program was seen as having a low priority in their workload. Midwives felt that the rapport between them and pregnant women was not sufficiently established at the first antenatal appointment to allow them to discuss alcohol issues appropriately. They reported that many women had already given up drinking or were drinking minimal amounts prior to the first antenatal appointment.
France et al.^33^ 2010 Australia	To identify the barriers that health professionals encounter in addressing alcohol use with pregnant women and to elucidate the strategies they use to overcome them.	53 health professionals (aboriginal health workers, allied health professionals, nurses, physicians) working in public and private health care in metropolitan and regional Western Australia	Qualitative design	Five focus groups and 19 in-depth interviews Thematic analysis	Health professionals identified strategies for obtaining alcohol use information from pregnant women, but they did not recognize moderate alcohol intake in pregnant women.
Jones et al.^34^ 2011 Australia	Explore the advice that midwives give to pregnant women about alcohol consumption and how this corresponds with the new NHMRC Australian guidelines to reduce health risks from drinking alcohol; and to understand what advice pregnant women believe they receive about alcohol consumption.	12 midwives from various sections in a large hospital, including prenatal, birth suite and postnatal/parent education and 12 pregnant women (not included in this study)	Qualitative study	Individual semi-structured interviews, face-to-face and phone Analyzed to identify common concepts and issue within and across the participants	Midwives and pregnant women consistently agreed that conversations about alcohol are generally limited to brief screening questions at the first visit, and the risks are not discussed or explained (except for high-risk women).
Loxton et al.^35^ 2013 Australia	To explore the views and experiences of women and service providers about their acquisition and utilization of information regarding alcohol use during pregnancy.	14 service providers (nursing, midwifery, social work, health work, telephone counselling, housing support, family support, and police) and 74 women (not included in this study)	Qualitative	Focus group interviews with HCP, semi-structured interviews with women Thematic analysis, including use of NVivo 8	Women and service providers expressed uncertainty about the alcohol recommendations were for pregnant women. Healthcare providers were inclined to discuss alcohol use with women they perceived to be high risk but not otherwise. Women felt pressure to both drink and not drink during their pregnancies. Those who drank discounted abstinence messages and reported a process of internal bargaining on issues such as the stage of their pregnancy and the type of beverages they consumed. Those who abstained did so mainly because they were afraid of being held responsible for any problems with their pregnancies or infants that might have occurred from drinking.
Oni et al.^36^ 2020 Australia	To explore midwives’ experiences of barriers and facilitators in antenatal settings to screening and referral of pregnant women who use substances.	Convenience sample of 18 midwives working in outpatient clinics and specialized units	Exploratory qualitative research design	Face-to-face and telephone interviews Semi-structured interview guide Thematic analysis based on qualitative methodology literature	Seven themes were derived under barriers: lack of validated screening tool; inadequate support and training; discomfort in screening; lack of multidisciplinary team; specialized treatment in regional and rural areas; workload and limited consultation time; and non- or partial disclosure of substance use, reluctance and non-adherence to referrals. Themes identified under facilitators: woman-centered philosophy of care, evidence of harm to neonates from substance use, experience and training, continuity of care, availability of multidisciplinary team and funding.
Pedruzzi et al.^37^ 2020 Australia	To identify the workforce development needs for prevention of PAE (prenatal alcohol exposure) by AOD (alcohol and other drugs) services in Newcastle, New South Wales (NSW), Australia.	26 participants, including nurses/midwives/gynecologists/social workers/psychologists/counsellors/program managers	Qualitative Based within a constructivism interpretivism paradigm and guided by grounded theory methods	Semi-structured research questions Thematic analysis	Three key themes were described: 1) Substance use during pregnancy: a complex issue; 2) Approaches to treatment; and 3) Service delivery: plugging the gaps.
Petersen Williams et al.^38^ 2015 South Africa	To investigate the healthcare providers’ perceptions of the acceptability and feasibility of providing SBIRT (screening, intervention, referral to treatment) to address substance use among pregnant women attending antenatal care in South Africa.	Female nursing and counselling staff (43) working at 2 midwife obstetric units, and those who would potentially be involved in or affected by the implementation of an intervention	Qualitative Consolidated framework for implementation research was used as a theoretical framework for the study	Individual interviews An open-ended, semi structured interview schedule guided the interviews Framework analysis	There is a substantial need for screening, brief intervention and referral to treatment for substance use among pregnant women. Such services could potentially be integrated into routine care. Several barriers that could hinder successful implementation of antenatal services were described.
Schölin et al.^39^ 2021 UK	To explore how UK midwives have implemented the guidelines and their views on advising women in line with the recommendations for abstinence.	A convenience sample of 22 clinical midwives and midwives working within research and education	Qualitative, part of a mixed-methods study of implementation of the guidelines in antenatal care	Focus groups and individual telephone interviews Theoretical Domains Framework guided the interview guide Thematic analysis following Braun & Clarke’s framework	Three high-level themes were developed: 1) views on the guidelines, 2) communication with women, and 3) strategies in addressing alcohol use.
Schölin et al.^40^ 2019 Sweden/England	To explore perceptions and practices of providing alcohol advice to pregnant women among frontline midwives in England and Sweden.	7 midwives (mws) working at a local maternity service in Liverpool (4 community mws, 3 mws with specialist practice areas) 9 midwives working at local general practices in Örebro county	Qualitative	Semi structured interviews Interview guide based on existing literature Thematic analysis according to Braun & Clarke, visualized using thematic networks	Four key themes were identified: 1) pregnant women’s lifestyles, 2) promoting a healthy lifestyle, 3) antenatal practices, and 4) the midwifery role.
van der Wulp et al.^41^ 2013 The Netherlands	To explore: 1) the advice Dutch midwives give, and 2) the information Dutch pregnant women and partners of pregnant women receive about alcohol consumption in pregnancy.	Convenience samples of: 1) 10 midwives working in urban and rural practices 2) 25 pregnant women and 9 partners (not included in this study)	Qualitative The I-Change Model served as a theoretical framework	1) Individual interviews by telephone and face-to-face 2) Five focus groups and four interviews Semi-structured interview routing, questions based on concepts of the I-Change Model Qualitative content analysis	1) Midwives intended to advise complete abstinence, although this advice was mostly given when women indicated that they consumed alcohol. Midwives reported lacking good screening skills and sufficient knowledge about the mechanisms and consequences of antenatal alcohol use and did not involve partners in their alcohol advice. 2) The views of pregnant women and their partners were congruent to the findings in study 1. In addition, pregnant women and their partners considered midwives as an important source of information on alcohol in pregnancy, Partners were interested in the subject, had a liberal view on antenatal alcohol use and felt ignored by midwives and websites. Pregnant women indicated that they received conflicting alcohol advice from their health professionals.
Whitehead et al.^42^ 2019 Australia	To understand the experiences and contextual factors that influence the ability of midwives to provide appropriate support to women regarding alcohol and other substance use during pregnancy in the Queensland context.	A purposive sample of 11 midwives working with pregnant women in private and public healthcare	Qualitative Critical realist approach	Interviews (via phone and in person) following a semi-structured interview schedule Thematic analysis guided by Braun & Clarke	Five overarching contextual influences were described including: 1) patient-level factors, 2) provider/patient-level factors, 3) provider-level factors, 4) organizational-level factors, and 5) broader system-level factors.

Data from the primary studies in the matrix, along with the translations and our notes from the individual and team discussions constituted the basis for the final synthesis. Meta-ethnography has the potential to create metaphors to enable deeper understanding^24^. The synthesis process allowed themes to emerge and resulted in an overarching lines-of-argument model^24^. The translations of the findings of each primary study into each other encompassed all the translations into an overarching metaphor in an interpretative yet systematic process. We began with an assumption of reciprocity between the findings, i.e. incorporating all the findings in an analogous interpretation, moving on to check possible contradictions that emerged, and finally constructing a lines-of-argument synthesis^24^ in which we identified different aspects of our topic, moving beyond the original primary studies and probing the metaphor. This type of interpretative synthesis, including the use of metaphors, is a characteristic feature of meta-ethnography. The synthesis should accurately portray both the shared and unique findings of the included studies. GRADE CERQual^27^ (Supplementary file Part 2) was used to assess confidence in the evidence from the study findings. The tool comprises four components: methodological limitations, relevance, coherence, and adequacy of data. The CERQual assessment revealed moderate confidence in the themes.

## RESULTS

We included studies describing screening and counselling for AIP conducted in six different countries, between 2010 and 2021. Eight Australian studies included professionals working in private and public ANC services in urban and rural parts of the country. One Canadian study investigated healthcare students’ attitudes and recommendations regarding AIP. Three studies originated from different parts of the UK, including clinical midwives, midwifery leaders and midwives working with research and education. One study compared midwifery practice in Sweden and England. Finally, the last two studies were conducted in South Africa and The Netherlands. Altogether, 311 HCPs were included in the studies, with sample sizes varying from 10 to 53 participants. Five studies included midwives exclusively, and 11 studies included midwives and other HCPs. Two studies did not include midwives, and one study did not provide the number of midwives included. However, approximately 150 midwives were included in the sample, constituting the majority of the participants.

Most studies used thematic analysis, including some form of interview as a data collection method. They described midwives’ and other HCPs’ attitudes towards alcohol use in pregnancy, their knowledge of the effects of alcohol consumption, their perceptions of the acceptability and feasibility of implementing screening and their perceptions of facilitators and barriers to addressing alcohol use in pregnancy.

### The synthesis

**Figure 2:**
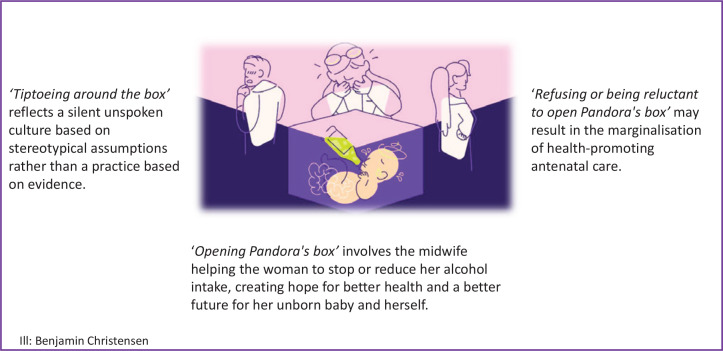
Pandora’s box

In the synthesis, we use the metaphor of Pandora’s box (Figure 2) to deepen our understanding of the topic. In Greek mythology, Pandora’s Box is related to the myth of Pandora, a woman whose curiosity led her to open a box that she was forbidden to open. When she removed the lid, all the troubles of the world swarmed out, never to be recaptured. Pandora was scared when she saw all the evil coming out and tried to close the box, without success. Only Hope was left in the box, stuck under the lid. In modern times, Pandora’s box has become an idiom referring to any source of great and unexpected trouble. Anything that looks ordinary but may produce unpredictable, harmful results can thus be called a Pandora’s box^28^.

Our findings show that many HCPs *‘tiptoe around the box’*, not wanting to face the consequences and responsibilities of asking women about their alcohol use. These HCPs argue that women do not drink during pregnancy or have stopped drinking. They perceive young partying women to be alcohol users and Muslim women to be abstinent. Some consider alcohol to be a problem only in early pregnancy, and question present recommendations to completely avoid AIP. Altogether, their practice reflects a silent unspoken culture based on stereotypical assumptions rather than a practice based on evidence.

Healthcare providers sometimes *‘refuse or are reluctant to open Pandora’s box’* and prioritize screening and counselling in other areas, seemingly due to lack of time. Further, routines for asking about AIP are absent or are only part of the booking visit. They believe that women have no interest in talking about AIP and try to avoid making women who have used alcohol feel guilty. Moreover, they seem to lack knowledge about AIP, display underlying doubts about whether alcohol use in pregnancy exists as a problem, and disregard the follow-up of alcohol use in antenatal care. This may result in the marginalization of health-promoting antenatal care.

The midwives and HCPs that eventually *‘open Pandora’s box’* understand the importance of establishing a trusting relationship with their patients to address the topic of alcohol use. They see the need for knowledge and screening tools, and they keep information at hand for women. In these cases, Hope is left in the box and may help create trust in the presence of the midwife, allowing the woman to share her fear, shame, worries, and health problems related to alcohol. Rather than closing Pandora’s box, the midwife listens to the woman and helps her plan how to stop or reduce her alcohol intake, creating hope for better health and a better future for her unborn baby and herself. An overview of themes and sub-themes is presented in Table 5.

**Table 5:** Themes, sub-themes and their references

*Themes*	*Sub-themes*	*References*
**Tiptoeing around Pandora’s box**	Doubts regarding the risk of AIP	29-32
	Prejudices related to women using AIP	29,33-35, 39
	Alcohol use as an accepted part of social life	29,30,33,35-37
	AIP is a health policy challenge	33,37
**Refusing to open Pandora’s box**	Controversy regarding responsibility to inform about AIP	31,32,34,36-41
	Informing about AIP is challenging	29,31-34,37-41
	Women neither want nor need information about AIP	29-31,33-35,37,39,41
	Women who drink during pregnancy do not disclose their alcohol consumption	29-32,35-39,41,42
	Providing information about AIP is low priority owing to time constraints and a lack of routines	30-32,34,38,40-42
**Opening Pandora’s box**	HCPs knowledge about AIP is fragmentary	30,31,33,36,37,39-42
	Guidelines and media present conflicting messages	30,32,33,36,37,39-42
	Screening for AIP seems to be haphazard, focusing on high-risk groups and uncertainties related to use	29,30,32,34-42
	A supportive, non-judgmental and sustained relationship makes it easier to discuss AIP with the women	29,31,32,35,37,40,42

### Tiptoeing around Pandora’s box 

*Doubts regarding the risk of alcohol use in pregnancy*

Healthcare providers considered small amounts of AIP harmless^29-31^. Some were skeptical that moderate use would pose a risk to the foetus^29,31^ and associated FASD with heavy, sustained drinking^31^. Uncertainty regarding the threshold at which alcohol could harm the baby prompted midwives to take a cautious stance in relation to advising abstinence^31^. Some argued that alcohol consumption in late pregnancy was less harmful than drinking in the first trimester^31^ while others supported the precautionary principle in the guidelines but acknowledged the uncertainty of risk of alcohol-related harm to the fetus at low levels of drinking^32^. Thus, the motivation to dissuade pregnant patients from occasionally drinking, if they were not considered at-risk, was low^29^. They believed that the recommendations to avoid alcohol consumption was introduced as a risk mitigation strategy and that evidence that small quantities of alcohol are harmful is lacking^30^.

*Prejudices related to women using AIP*

While some midwives believed that the risk of prenatal alcohol consumption crossed social strata^33^, others argued that AIP was related to socio-economic status^30,34^. They considered AIP to be a concern for vulnerable groups^29,34^ and argued that there were other underlying issues present, such as mental health problems^30^. Muslim women were considered non-drinkers^34^, and some HCPs found it difficult not to fall for the stereotype that women who drink during pregnancy come from lower income families^29^. It was important to understand the role of alcohol in a woman’s life, as some women might be affected by their own mothers’ substance use or by trauma or the previous removal of a child^35^. They considered alcohol-related harm to be linked to the problem of addiction or binge drinking^29^, and FASD was considered an issue particularly for families in which there were other issues associated with alcohol use^33^.

*Alcohol use as an accepted part of social life*

The attitude that it is acceptable to drink a little during pregnancy is widespread in society, and it informs women’s decisions about drinking^33^. The media, including social media, played an important role in women’s perception of AIP, and midwives believed that women felt social pressure to drink during pregnancy^30^ or felt that drinking was unproblematic due to the legal and social acceptance of alcohol^35^. Australia is considered a drinking culture and midwives’ advice was considered secondary to contrasting advice given by family and friends^36^. Midwives considered nine months a long time to abstain given the centrality of alcohol in many social situations^37^. It was challenging to discuss AIP in social settings^33^, and partners rarely considered changing their own behaviors to support future mothers^37^. Thus, spouses should also be encouraged to limit their alcohol consumption^29^.

*Alcohol in pregnancy is a health policy challenge*

Healthcare providers questioned the reluctance to highlight health risks related to AIP^33^. They believed that alcohol-related harm was minimized in public discourse because of politicians’ personal views on and experiences with alcohol, as well as political and economic pressure on the government by the alcohol industry^33^. Drinking in general, and women’s drinking in particular, is a public health concern, and alcohol consumption is a massive issue^37^. Communication problems around alcohol use in pregnancy could be related to broader social patterns of alcohol use^33^.

### Refusing to open Pandora’s box 

*Controversy regarding the responsibility to inform about alcohol in pregnancy*

Screening for substance use in pregnancy was part of midwives’ role, and some felt confident discussing AIP with their clients^31,37-40^. Midwives who did not drink alcohol were strongly against AIP, while midwives who did drink alcohol were skeptical of advising abstinence^31^. Some argued that advising women about AIP was ‘none of their business’^36^ while others felt disinclined to discuss alcohol use because of personal beliefs^36^. Often, they advised women to abstain from alcohol during pregnancy^31,32,37,39,41^. However, some did not address moderate consumption^34^ or recommended not to drink alcohol except on special occasions^39^. Midwives should provide evidence-based information^32^, and one midwife called for clarity from the government to explicitly state that abstinence is the safest choice and for all HCPs to give the same advice^37^.

*Informing about alcohol use in pregnancy is challenging*

Some midwives and other HCPs found it difficult to ask questions about AIP^32,33^. Others considered the topic sensitive but not difficult to raise with clients^37^. Midwives argued that recommending abstinence can lead women to develop feelings of guilt or anxiety if they have unwittingly consumed AIP^34,39^. Thus, alleviating anxiety about unintended exposure to alcohol before the woman knew she was pregnant was important^32^. According to one midwife, if a client were to disclose that she had had a drink on a special occasion, she would alleviate her fears but continue to advise no alcohol^41^. However, it was important to have good rapport with the women^34,38^ as asking about AIP could appear stigmatising^29^ or ‘prying’^41^. Thus, midwives had to be careful not to alienate the women from antenatal care when discussing sensitive alcohol issues^31,34^, as stigma created a threat of disengagement^38^. In this regard, sometimes harm reduction was a more beneficial approach^29,40^.

*Women neither want nor need information about alcohol use during pregnancy*

There was a widespread belief among midwives that very few women drink during pregnancy^34,37,41^ or that they stop drinking once their pregnancy is confirmed^29-31^. Pregnancy worked as a motivator to stop drinking^35^, and women knew not to drink or to drink minimally^34^. Consequently, some did not ask directly about AIP as they were convinced that their clients already abstained from alcohol^34,39^. Further, the use of AIP raised the issue of women’s autonomy over their own bodies and the rights of fetuses versus the rights of women^33^. Some argued that women who are ‘well-off’ are aware of risks and handle their consumption^29^, but at the same time, midwives reported that they were aware that some alcohol or specific drinks were considered acceptable among women of higher socioeconomic status^37^. Thus, recommendations and prenatal care would be different for ‘well-off’ women and ‘at-risk’ women^29^.

*Women who drink during pregnancy do not disclose their alcohol consumption*

According to midwives and other HCPs, some women were not truthful when they claimed to abstain from AIP^29,39,41^. Women knew that they should not drink^31^, but midwives were uncertain if this really stopped them from drinking^30^. Thus, women could be recovering alcoholics or binge drinkers without HCPs being aware^29^. They believed that the underreported alcohol intake was related to the stigma around AIP^37^, arguing that women did not disclose if they were drinking^32^. Some were not ready to disclose^38^ while others were unwilling to disclose^35^, or they would ‘bargain’ about alcohol use^36^. Non-disclosure or partial disclosure was associated with a lack of knowledge about risks and feelings of guilt and shame about use^38^. Other reasons not to disclose were a lack of continuity of care and insufficient rapport with the care provider^38^. Thus, lack of honesty and willingness to disclose were barriers to getting help^42^.

*Providing information about alcohol in pregnancy is low priority owing to time constraints and a lack of routines*

Screening for AIP added to midwives’ workload^31,34,38,40,42^, resulting in alcohol consumption only being discussed at the booking visit^41^. Alcohol intervention was given low priority^31,34,40^ as there was ‘so much other stuff’ to handle^30^. Also, midwives needed to prioritize other issues that were more pressing^32^. The short duration of pregnancy restricted midwives’ ability to establish adequate support to enable women to reduce or cease alcohol consumption^34^. Nevertheless, midwives’ knowledge and understanding of the risks of AIP influenced the level of priority, and practices and procedures played an important role in providing information^31^.

### Opening Pandora’s box 

*Healthcare providers’ knowledge about alcohol in pregnancy is fragmentary*

Most midwives had a good understanding of problems related to AIP^30^ and were aware that alcohol use could cause birth defects^31,37^. Some were able to discuss the consequences of AIP for the fetus and for reproductive health^42^, but misconceptions were also reported^39^. Some were aware of FAS but could not explicitly describe the condition^30,39^ or believed it was caused only by high alcohol consumption^36^. Lack of knowledge about the risks of alcohol^33^ or the effects of alcohol on infants^41^, including knowledge about FASD, was also described^40^. HCPs stated that they had sufficient knowledge to deal with AIP appropriately^30,42^ but lacked the knowledge to fully support their clients^42^ and wondered whether telling women they could have one or two drinks would encourage them to drink more^29^. They were reluctant to engage too deeply with women’s substance-use problems if they did not know how to handle the information^42^, and some had no plans to look for additional information, although they were aware that additional knowledge would allow them to give better advice^39^.

*Guidelines and the media present conflicting messages*

Some midwives were unaware of the guidelines regarding alcohol consumption, and many described a need for adequate materials to give to their clients^42^ to educate them about AIP^40^. In addition to verbal advice, they used different sources of information including written^39,41^ and digital^41^. They described challenges related to the use of recommendations as information and guidance were sometimes conflicting^32^ and expressed a lack of clarity about recommendations^36^. Furthermore, changing guidelines and conflicting information in the media caused confusion about safe levels of AIP^37^ and not all midwives were aware of the current official recommendations^32,39^. Information about AIP was lacking in the media^30^, and some midwives found that the media presented mixed messages about AIP^33^. In addition, their clients would receive conflicting information and advice from relatives and friends, causing confusion about safe levels of AIP^37^.

*Screening for alcohol in pregnancy seems to be haphazard, focusing on high-risk groups and uncertainties related to use*

Midwives’ sense of responsibility and willingness to help and reduce harm was a strong facilitator to screening^38^, but the way AIP was assessed varied^32^. Routine screening was important^42^ and generally took place in early pregnancy^30,37,38,40,41^. If nothing came up at the first visit, women were not asked again^30,37^. Further, the quantity and frequency of consumption was not always investigated^30^. Asking all women about AIP rather than focusing on highrisk groups normalized the topic^34,37^, and when questions were part of routine practice, they could easily be followed up^34^. For some HCPs, the decision to screen was based on personal feelings about and perceptions of women in their care^29^. Midwives argued that they would talk with their clients about AIP if the clients requested the information or if there were obvious signs of alcohol abuse^30^, when clients admitted alcohol use^39^, or if they perceived the woman to be at risk of using AIP^36,41^. They were generally able to identify women who drank high levels of alcohol^34^. However, supporting heavy users was difficult^34^. Some referred women with alcohol problems to specialist clinics^41^ but described a lack of multidisciplinary care and specialized treatment^38^. Having an appropriate and validated screening tool was important for midwives to enhance the effectiveness of screening^38^ and some used screening tools as part of their assessment for sensitive topics^40^, for example, the AUDIT screening questionnaire^32,37^. The questionnaire was sometimes used in person during appointments, or it was sent to women ahead of their appointments^32^. One study reported involving partners in screening^37^. Some providers did not use tools at all^38^ while others used a directive style of counselling^42^ or a strategy to involve women in a discussion about their current drinking habits^32^. The importance of a non-judgmental and supportive approach, as well as a causal and conversational assessment style was underlined^40^. Discussion about AIP should be non-stigmatising^35^ and non-confrontational, making disclosing comfortable^32^. Some midwives mentioned the value of having a checklist with questions about AIP^34^ as well as knowledge about evidence-based strategies to support women^40^. Experience and training on how to initiate a conversation about alcohol in a non-judgmental manner were considered facilitators to screening for AIP^38^. However, midwives described having limited^36^ or no formal training or education about substance use screening^38,40,42^. Some had received training in motivational interviewing^32,37^, but learning was also selfdirected through Internet searches, reviewing of articles and informal information sharing^36^. Some had learned about AIP during their midwifery training^39^, through voluntary extra courses, or from the media^39^, but many expressed a strong desire to learn more about effective ways of responding to the problem^42^, arguing that a lack of skills and resources prevented them from raising the subject^34^.

*A supportive, non-judgmental and sustained relationship makes it easier to discuss alcohol in pregnancy with women*

Pregnancy was believed to motivate changes in health behaviour^31^. Thus, it was important for providers to build a strong relationship with their clients based on an empathic, non-judgmental approach^29,35,37^. Establishing and maintaining relationships facilitated trust, and women were more likely to disclose sensitive information, seek support, and be receptive to information and advice from a midwife with whom they had a trusting relationship^32,40^. Screening for AIP should take place later in pregnancy, when the trusting relationship is established, to make it easier for the woman to disclose^32^. Screening at the first appointment may not be appropriate because of its potential impact on the provider–client relationship^31^. Women should be allowed to feel supported rather than judged^29^, and advice and guidance should be tailored to their individual situation^37^. Thus, attitudes towards women who used AIP were important as women were only likely to disclose if they had a good rapport with their midwife and encountered persons with a non-judgmental attitude^42^.

## DISCUSSION

The synthesis shows that screening and counselling for alcohol use in pregnancy was a challenging issue. Some midwives employed strategies to avoid opening a Pandora’s box while others turned their backs to the box or left the cover ajar in an effort to minimize the troubles emanating from use of alcohol in pregnancy. Fortunately, some had the courage to remove the cover and deal with the challenges related to AIP, finding troubles but also hope.

According to Popova et al.^4^, nearly 10% of women who used to drink alcohol before they became pregnant continue drinking in pregnancy. Thus, it is important to screen all women for AIP, not just women considered to be high-risk. Most HCPs were aware of the recommendations to abstain from AIP; however, uncertainties regarding the threshold at which alcohol use could harm a baby made it difficult to advise abstinence^31^. Some providers associated FASD with heavy sustained drinking or other issues associated with alcohol^31^, while others doubted the risks of AIP^29-31,39^ and demonstrated an attitude of accepting AIP in certain social circumstances. Studies exploring women’s attitudes towards AIP demonstrate that exceptions to abstinence generally occur during specific social occasions^43^. This implies that in some societies, moderate consumption of AIP is socially acceptable^44,45^ although it conflicts with recommendations to abstain^35^. Nevertheless, HCPs considered AIP a public health concern and questioned society’s reluctance to highlight health risks related to AIP. They argued that alcohol-related harm was minimized in the public discourse because of politicians’ personal views about alcohol, but also by political and economic pressure on the government by the alcohol industry^33^. Similar results were found in a study by Lim et al.^46^ in which they argue that ‘alcohol industry corporate social responsibility bodies may use strategic ambiguity and other informational tactics to “nudge” women toward continued drinking in pregnancy to protect the female alcohol market’. For some midwives, minimizing anxiety and guilt for women who had consumed alcohol before they knew they were pregnant was important^41^; for others, discussing AIP with women was not considered part of their role^36^. Similar findings have been described by general practitioners (GPs)^47^. A Swedish study found that young women were familiar with recommendations to abstain from AIP, but they had heard that consuming small amounts of alcohol was harmless^44^. This underlines the importance of midwives bringing up the topic in early antenatal care consultations. In this regard, updated evidence-based knowledge about risks related to the use of AIP, as well as knowledge about how and when to screen for AIP, is vital. It is therefore discouraging that most midwives described having limited formal training or education about substance use screening^38,40,42^. Instead, learning was self-directed through courses, Internet searches, article reviews and informal information sharing^36^. However, they expressed a strong desire to learn more about effective ways of responding to the problem^42^.

Public stigma related to alcohol use in pregnancy is intense^48^ and consumption of AIP is often judged harshly^49^. A recent attitude concerning AIP has raised the issue of women’s autonomy over their own bodies, as well as the rights of the fetus versus that of the woman^33^. Recent research also highlights a new phenomenon, the so-called ‘wine mom’ culture, where shared norms and beliefs about alcohol and motherhood are spread through social media^49^. Harding et al.^50^ state that wine consumption represents modern motherhood; it is socially accepted as self-care and serves as an act of active resistance against the traditional mother role and the expectations of motherhood. Skagerstrøm et al.^44^ found that some women advocating autonomy argued that drinking small amounts of AIP was acceptable. However, research is needed to explore to what extent this attitude is reflected among pregnant women in general. Nevertheless, the attitude contradicts stereotypical assumptions that alcohol use is related to vulnerable groups of women and underscores the importance of considering sociocultural understandings as well as evidence-based information when assessing and counselling women for AIP.

Our findings demonstrated a widespread belief among midwives and other HCPs that very few women drink during pregnancy^34,37,41^ or that they stop drinking once their pregnancy is confirmed^29-31,34,35^, with pregnancy working as a motivator to stop drinking. Health promotion is a key issue in antenatal care, and midwives and other HCPs caring for pregnant women have a responsibility to include planned interventions to improve health^51^. Nevertheless, initiating a dialogue about alcohol comes with new problems that might be difficult to handle as knowledge of AIP is fragmentary, guidelines and the media present conflicting messages, and screening for AIP seems to be haphazard, focusing on highrisk groups and uncertainties related to screening strategies and training^19^. However, midwives’ sense of responsibility and willingness to help and reduce harm is seemingly a strong facilitator of screening. For some, having an appropriate and validated screening tool was important to enhance the effectiveness of screening^32,37^. Others used a directive style of counselling^42^ or involved women in a discussion about their current and pre-pregnancy drinking habits^42^. The use of a non-judgmental and supportive approach, as well as a causal and conversational assessment style, was important to make screening for AIP non-stigmatizing and non-confrontational and disclosing comfortable^35,38^. Equally important was establishing relationships with women to facilitate trust^32,40^. Women were more likely to disclose sensitive information to, seek support from, and be receptive to information and advice from a midwife with whom they had a trusting relationship. Although many providers advocated early screening^30,37,38,40,41^, some argued that screening should take place later in pregnancy, when a trusting relationship had been established^32^. Morrello et al.^52^ found that midwives who received training in screening and counselling women for AIP increased use of screening at first antenatal contact, including using validated tools and making multiple enquiries. The midwives argued that by introducing information early, it was possible to reduce alcohol-related harm and that building rapport quickly with their clients was part of being a midwife. Further, using a tool demonstrated to women that screening was universal and a standard part of practice^52^. Brief interventions (BI), such as evidence-based counselling sessions where the midwife seeks to promote behavioral change, are recommended by the WHO^18^. These are often paired with proactive screening, and can help women make healthy choices. In the ANC context, a BI may strengthen the relationship between a woman and her midwife while simultaneously advancing the public health message that abstaining from AIP is necessary to prevent fetal harm. Simultaneously, the use of BIs, which involve reaching out to women in a client-centered way rather than offering a pre-set list of topics to be covered in ANC, may enhance the possibility of disclosure. However, given the stigma related to the use of alcohol in pregnancy, it is imperative that midwives and other HCPs demonstrate a non-judgmental attitude when screening for AIP as some women may feel the abstinence message to be paternalistic and confrontational. Likewise, more attention should be given to the importance of including the woman’s partner in screening and counselling for AIP.

### Strength and limitations

This meta-ethnography synthesizes midwives’ and other HCPs’ experiences with screening and counselling for AIP. A strength of the study is the rigorous methodology of systematic literature searches employed in 2021 and 2023. Altogether, more than 6000 articles published between 2010 and 2023 were screened. However, only 14 articles satisfied the inclusion criteria, demonstrating that qualitative research about AIP is scarce. We were also surprised to find that research on AIP in the Nordic countries was mainly quantitative. Our sample included 311 midwives and HCPs from six different countries, who pointed to challenges related to screening and counselling on different levels, in different cultures and policies and healthcare systems. Thus, it provided empirical variation and sufficient data to achieve the study aim. We are aware that we may have left out studies, that our keywords may not have been comprehensive, and that the inclusion criteria may have resulted in the loss of relevant information. Still, the use of e-MERGe meta-ethnography reporting guidance provided transparency and reflexivity to the process, and the use of the CERQual Qualitative Evidence Profile contributed to transparency and trustworthiness. Another strength is that our research team consisted of three people with different healthcare backgrounds (public health nurse, psychiatric nurse, midwife) and expertise in the subject under study (alcohol use, antenatal care, public health nursing) as well as expertise in methodology (qualitative research, metasynthesis).

## CONCLUSIONS

Our lines-of-argument synthesis suggests that screening and counselling for alcohol use in pregnancy is challenging and deserves more attention. Healthcare providers described a lack of knowledge and appropriate screening tools but also mentioned conflicting messages regarding the use of guidelines and the understanding that social drinking is acceptable in pregnancy. Healthcare education has the important task of ensuring that healthcare personnel have sufficient evidence-based knowledge to handle the situation. Increased alcohol use and ‘wine mom’ culture challenge the general understanding that pregnancy is a motivator to stop drinking and require new strategies to advocate abstinence. In the future, a health-promoting, tailored approach offering women sufficient evidence-based information in pre-pregnancy and early pregnancy should be implemented.

## Data Availability

Data sharing is not applicable to this article as no new data were created.
