# Supplementary material for: Opening Pandora’s box: A meta-ethnography about alcohol use in pregnancy from midwives’ and other healthcare providers’ perspectives
Source: Eur J Midwifery. 2023 Jul 3;7:14. doi: 10.18332/ejm/166189 (PMC10316433; doi:10.18332/ejm/166189)
Supplement: Supplementary file 1 [file EJM-7-14-s1.pdf]

SUPPLEMENTARY FILES

Supplementary file 1. Literature searches 2021 and 2023

Database: **Maternity & Infant Care**
Dato: *01.02.2021*
Antall treff: *1434 – fjernet duplikater, 1411*
Kommentar:

#	Searches	Results
9	Limit to yr="2010 -Current"????	1434
8	6 AND 7	3316
7	3 OR 4 OR 5	4004
6	1 OR 2	157406
5	alcohol*.mp	4004
4	Alcohol consumption.de	1
3	(Alcohol or Alcohol drinking).de	1350
2	pregnan* OR maternal* OR Gestation.mp	157406
1	Pregnancy.de	60288

Database: **Maternity & Infant Care**
Dato: *17.01.2023*
Antall treff: ***233 (229 – fire duplikater)***
Kommentar:

#	Searches	Results
9	Year 2021-2023	233
8	6 AND 7	3674
7	3 OR 4 OR 5	4381
6	1 OR 2	182228
5	alcohol*.af	4381
4	Alcohol consumption.de	1
3	(Alcohol or Alcohol drinking).de	1388
2	pregnan* OR maternal* OR Gestation.af	182228
1	Pregnancy.de	62863

Database: Cinahl
Dato: 01022021
Antall treff: 3442 - 3301 etter fjernet dubletter
Kommentar:

#	Searches	Results
9	Limiters - Published Date: 20100101-20211231; Peer Reviewed	3442
8	6 AND 7	6008
7	4 OR 5	109 669
6	1 OR 2 OR 3	309 469
5	Alcohol*	109 323
4	MH “Alcohol drinking+”)	31 959
3	pregnan* OR maternal* OR Gestation	301,212
2	Pregnan*	251 934
1	MH "Pregnancy+")	220 714

Database: Cinahl
Dato: 17.01.23
Antall treff:
Kommentar:

#	Searches	Results
10	Date of Publication:February 2021-december 2023 ; Scholarly (Peer Reviewed) Journals	84
10	7 AND 8 AND 9	1,001
9	5 OR 6	436,726
8	3 OR 4	123,449
7	1 OR 2	351,763
6	qualitative research or qualitative study or qualitative methods or interview	421,149
5	(MH "Qualitative Studies+")	177,485
4	Alcohol*	123,015
3	MH “Alcohol drinking+”)	35,660
2	pregnan* OR maternal* OR Gestation (All text)	342, 293
1	MH "Pregnancy+")	246,730

Database: Medline
Dato: 12.08.2021
Antall treff: 1075 (1 duplikat)
Kommentar:

#	Searches	Results
10	Limiters - Published Date: 20100101-20211231; Peer Reviewed	1076
10	7 AND 8 AND 9	1,759)
9	5 OR 6	382,094
8	3 OR 4	465,448
7	1 OR 2	859,957
6	TX qualitative research or qualitative study or qualitative methods or interview	381,981)
5	(MH "Qualitative Research+")	64,406)
4	TX Alcohol*	465,273
3	MH “Alcohol drinking+”)	60,171
2	TX pregnan* OR maternal* OR Gestation (All text)	851,551
1	MH "Pregnancy+")	578 805

Database: Medline
Dato: 17.01.2023
Antall treff: 139
Kommentar:

#	Searches	Results
10	Limiters - Published Date: 20210201-202231231; Peer Reviewed Journals	139
10	7 AND 8 AND 9	1,456
9	5 OR 6	466,978
8	3 OR 4	522,391
7	1 OR 2	1,300,893
6	TX qualitative research or qualitative study or qualitative methods or interview	466,821

5	(MH "Qualitative Research+")	78,818
4	TX Alcohol*	522,065
3	MH “Alcohol drinking+”)	76,754
2	TX pregnan* OR maternal* OR Gestation (All text)	1,287,652
1	MH "Pregnancy+")	988,276

Database: Scopus
Dato: 13.08.2021
Antall treff: 236 (en duplikat)
Kommentar:

#	Searches	Results
6	LIMIT-TO (Article + review)	237
5	LIMIT-TO (PUBYEAR 2010-2021)	241
4	1 AND 2 AND 3	304
3	Article title, abstract, keyword (qualitative studies or qualitative research or qualitative study or qualitative methods or interview)	417,582
2	Article title, abstract, keyword (Alcohol*)	940,095
1	Article title, abstract, keyword (pregnan* OR maternal* OR gestation)	1,393,778

Database: Scopus
Dato: 17.01.2023
Antall treff: 282 (en duplikat)
Kommentar:

#	Searches	Results
6	LIMIT-TO (source type=Article + review)	80
5	LIMIT-TO (PUBYEAR 2021-2023)	82
4	1 AND 2 AND 3	373
3	Article title, abstract, keyword (qualitative studies or qualitative research or qualitative study or qualitative methods or interview)	500,482
2	Article title, abstract, keyword (Alcohol*)	1,015,628

1	Article title, abstract, keyword (pregnan* OR maternal* OR gestation)	1,490,544

Supplementary file 2. CERQual Qualitative Evidence Profile

**Review finding**		**Studies contribu-ting to the review finding**	**Assessment of methodological limitations**	**Assessment of relevance**	**Assessment of coherence**	**Assessment of adequacy**	**Overall CERQual Assessment of confidence**	**Expla-nation of judgement**
Tiptoeing around Pandora's box	Doubts regarding the risk of alcohol use in pregnancy<br><br>Prejudices related to women using AIP<br><br>Alcohol use as an accepted part of social life<br><br>Alcohol in pregnancy is a health policy challenge	29,30,31,32<br><br>29,39,33,34, 35<br><br>29,30,33,35, 36,37<br><br>33,37	Minor methodological considerations.<br><br>All studies but two lacked clarity about reflexivity.<br><br>Two studies had minor concerns about data analysis.	Minor concerns about relevance.	Minor concerns regarding coherence.<br><br>Data reasonably consistent within and across studies.	Moderate concerns as the data were partially rich.	Moderate confidence.	The finding was graded as moderate confidence because of minor methodological considerations, minor concerns about relevance and coherence and moderate adequacy of data.
Refusing to open Pandora's box	Controversy regarding the responsibility to inform about alcohol in pregnancy<br><br>Informing about alcohol use in pregnancy is challenging<br><br>Women neither want nor need information about alcohol use in pregnancy	31,32,34,36, 37,38,39,40, 41<br><br>29,31,32,33, 34,37,38,39, 40,41<br><br>29,30,31,33, 34,35,37,39, 41	Minor methodological considerations. All but four studies lacked clarity about reflexivity.<br><br>Three studies had minor concerns about data analysis.	Minor concerns about relevance.	Minor concerns regarding coherence. Data reasonably consistent within and across studies.	Minor concern about adequacy of data. One study provided 'thin' data.	Moderate confidence.	The findings were graded as moderate confidence because of minor methodological limitations, minor concerns about relevance and coherence and minor concern about data adequacy in one study.

	Women who drink during pregnancy do not disclose their alcohol consumption<br><br>Providing information about alcohol in pregnancy is low priority owing to time constraints and a lack of routines	29,30,31,32,35,36,37,38,39,41,42<br><br>30,31,32,34,38,40,41,42						
Opening Pandora's box	Healthcare providers' knowledge about alcohol in pregnancy is fragmentary<br><br>Guidelines and media present conflicting messages<br><br>Screening for alcohol in pregnancy seems to be haphazard, focusing on high-risk groups and uncertainties related to use<br><br>A supportive, non-judgemental and sustained relationship makes it easier to discuss alcohol in pregnancy with women	30,31,33,36,37,39,40,41,42<br><br>30,32,33,36,37,39,40,41,42<br><br>29,30,32,34,35,36,37,38,39,40,41,42<br><br>29,31,32,35,37,40,42	Minor methodological considerations. All but four studies lacked clarity about reflexivity.<br><br>Three studies had minor concerns about data analysis.	Minor concerns about relevance.	Minor concerns regarding coherence.	Minor concerns about adequacy of data. One study provided 'thin' data.	Moderate confidence.	The findings were graded as moderate confidence because of minor methodological limitations and minor concerns about relevance and coherence.

Definitions of levels of confidence from the CERQual evaluation:
High confidence: It is highly likely that the review finding is a reasonable representation of the phenomenon of interest.
Moderate confidence: It is likely that the review finding is a reasonable representation of the phenomenon of interest.
Low confidence: It is possible that the review finding is a reasonable representation of the phenomenon of interest.
Very low confidence: It is not clear whether the review finding is a reasonable representation of the phenomenon of interest.

© 2023 Dahl B. et al.
